# Impact of Sense of Coherence on Oral Health Behaviors: A Systematic Review

**DOI:** 10.1371/journal.pone.0133918

**Published:** 2015-08-14

**Authors:** Maryam Elyasi, Lucas Guimarães Abreu, Parvaneh Badri, Humam Saltaji, Carlos Flores-Mir, Maryam Amin

**Affiliations:** 1 Division of Pediatric Dentistry, School of Dentistry, University of Alberta, Edmonton, Canada; 2 Pediatric Graduate Program, School of Dentistry, Federal University of Minas Gerais, Belo Horizonte, Brazil; 3 Visiting Researcher, School of Dentistry, University of Alberta, Edmonton, Canada; 4 Orthodontic Graduate Program, School of Dentistry, University of Alberta, Edmonton, Canada; National Cardiovascular Center Hospital, JAPAN

## Abstract

**Objectives:**

The aim of this review was to critically analyze the empirical evidence on the association between Sense of Coherence (SOC) and oral health behaviors through a systematic approach.

**Methods:**

A systematic search up to April 2015 was carried out using the following electronic bibliographic databases: PubMed, Ovid MEDLINE; ISI Web of Science; and Ovid PsychInfo. Studies were included if they evaluated the relationship between SOC and oral health behaviors including tooth cleaning, fluoride usage, dietary habits, dental attendance, and smoking. We excluded studies that only assessed the relationship between oral health status and SOC without evaluating oral health behaviors. The New Castle Ottawa (NOS) quality assessment checklist was employed to evaluate the methodological quality of included studies.

**Results:**

Thirty-nine potential papers met the preliminary selection criteria and following a full-text review, 9 papers were finally selected for this systematic review. Results provided by the included studies indicated different levels of association between SOC and oral health behaviors. The most frequent behaviors investigated were tooth brushing and dental attendance pattern. The impact of SOC on performing positive oral health behaviors, to some extent, was related to demographic and socio-economic factors. In addition, mothers’ SOC influenced children’s oral health practices.

**Conclusions:**

A more favorable oral health behavior was observed among those with a stronger SOC suggesting that the SOC can be a determinant of oral health-related behaviors including tooth brushing frequency, daily smoking, and dental attendance.

## Introduction

During the last three decades, theoretical approaches have been introduced to the public health literature to explain the importance of social context and its association with biological and psychological determinants of health and illness. [1,2] For instance, public health-related studies have identified “stress” as an important social determinant of patient’s adherence to medical advice and health promotion programs. [3,4] Consequently, the “Salutogenic” theory, which is a social health-related theory, was developed to explain the correlations between health, stress, and coping. [3] This theory, therefore, emphasizes the role of psychosocial determinants in maintaining human well-being rather than causing the diseases. [5]

Sense of coherence (SOC) is the main constituent of the Salutogenic theory. It evaluates the individual’s capability to use existing resources in order to overcome difficulties and cope with life stressors to perform healthy behavior and stay well. [3,5] People with stronger SOC can better cope with existing stressors in their social life. [6] This enables them to benefit from an increased feeling of well-being. [1,3,6] SOC is a cross-cultural concept that is not influenced by age, sex, ethnicity, nationality, and study design. [5] It relies on the following three key competencies: comprehensibility, manageability and meaningfulness. [3,5] These competencies have dynamic interactions. [3,6] It means that people who have higher level of SOC are more capable of perceiving typical stressors coming from their society (e.g., racial segregation, employment rate, and family relationships) [7] and environment (e.g., housing condition, traffic patterns, and environmental tobacco smoke) [7] as non-stressors (comprehensibility). They are also able to efficiently utilize available resources to control stressful circumstances (manageability), and to cope with stressors by having more enthusiasm, intention, and dedication (meaningfulness). [5] Thus, in people with a stronger SOC, there is a higher expectation for a superior health status and quality of life with fewer symptoms in case of existing illness. [8, 9]

A strong association between higher SOC and lower incidence of chronic diseases [10, 11] and better quality of life [9,12,13] has been reported in several studies. In the field of oral health, the incidence of chronic oral diseases such as dental caries and periodontitis is not only related to biological factors, but may also be influenced by non-biological factors such as oral health behaviors. [14, 15] An individual’s behavior is the manifestation of several determinants such as psychosocial and environmental factors that can be influenced by the SOC concept. [16, 17] This concept is considered as a practical model emphasizing the psychosocial aspect of oral health promotion rather than the risk of the disease. [3] SOC has also been considered as a psychosocial determinant of oral health behaviors in adults. [17, 18] In other words, individuals who have a stronger SOC are more intended to attend regular dental check-ups, [16] clean their teeth more often, [19] and have healthier dietary habits [18] as compared to their counterparts who have lower levels of SOC. In addition, oral health perceptions as well as oral-health related quality of life (OHRQoL) of both children and adolescents are significantly affected by their parents' SOC. [20, 21] However, a few related studies failed to report any specific association between SOC and some oral health behaviors (e.g., frequency of sugar intake and tooth brushing) [16,22] or OHRQoL in different age groups. [23]

To the best of our knowledge, there is no critical analysis in the dental literature that has attempted to summarize the existing evidence regarding the association between the SOC and oral health-related behaviors. The purpose of this article is, therefore, to critically analyze the association between SOC and oral health behaviors by a systematic review of the available data. This critical appraisal of evidence is needed to update the oral health community about the impact of SOC on oral health behaviors, which may ultimately help with developing effective oral health promotion strategies and interventions when the SOC is considered as a variable of interest in the future studies.

## Materials and Methods

This systematic review is reported, whenever applicable, in accordance with the PRISMA (Preferred Reporting Items for Systematic Reviews and Meta-Analysis) statement checklist. [24]

### Protocol and registration

This systematic review’s protocol was not registered in advance.

### Eligibility criteria

We included studies in the domain of oral health that evaluated the relationship between SOC and oral health behaviors including tooth cleaning, fluoride usage, dietary habits, dental attendance, and smoking. No restrictions were applied regarding language, study design, age, sex, culture or socio-economic status. We excluded studies that only assessed the relationship between SOC and oral health status without evaluating the impact of SOC on oral health behaviors. The reviews of the literature, meeting abstracts, editorial letters and qualitative studies were excluded.

### Data sources and search strategy

Comprehensive searches up to April 1, 2015 were carried out using the following electronic bibliographic databases: PubMed (1966 to April 2015, week 1), Ovid MEDLINE (1980 to 2015, week 12); ISI Web of Science (1965 to April 1, 2015); and Ovid PsychInfo (1980 to April 2015).

The search strategy was designed with the assistance of a health sciences librarian. Keyword and their combinations were first chosen and used in PubMed (Table 1). Then, the terms were adapted to run the search in other databases (S1 Appendix). Hand searches were made on the reference lists of the selected articles for any potential papers not identified through the electronic search. A partial gray literature search was finally performed by using Google Scholar and Google search engine.

**Table 1:** Search Strategy (in PubMed).

**#1** "sense of coherence"[All Fields] OR "sense of coherence scale"[All Fields]) OR "salutogenic model"[All Fields]) OR "salutogenic approach"[All Fields]) OR "salutogenic theory"[All Fields]) OR "salutogenic concept"[All Fields])
**#2** ("Oral Health"[All Fields] OR "oral hygiene"[All Fields]) OR "tooth brushing"[All Fields]) OR "dental attendance"[All Fields]) OR "dental education"[All Fields]) OR "dental"[All Fields]) OR "dentistry"[All Fields]) OR ("dental caries"[MeSH Terms] OR ("dental"[All Fields] AND "caries"[All Fields]) OR "dental caries"[All Fields] OR "caries"[All Fields])) OR "oral habit"[All Fields])
**#3 #1 AND #2**

### Study selection

The study selection was performed in two phases. In phase one, three reviewers (M.E, L.A, P.B) independently evaluated the list of the titles and abstracts of potentially articles to be included. If the abstract was judged to contain insufficient information for a decision of inclusion or exclusion, the full article was obtained and reviewed before a final decision was made.

For phase two, the full texts of potentially relevant abstract were retrieved and the selection criteria were applied again to confirm the final selection. Discussion between reviewers regarding any inconsistency in the inclusion of articles was done until agreement was reached. As a third reviewer was involved when discrepancies arose, the final agreement was of 100%.

### Data extraction and data items

Data were extracted from each of the selected articles separately by the same three examiners on the following items: author and year of publication, study design and aim of the study, demographic characteristics, oral health behaviors, and SOC scale used, sampling, statistical analysis and the main findings of the study were also collected and summarized (Table 2). Disagreements between investigators were resolved by reexamining the studies until consensus was reached. In the case of any missing information or uncertainty in evaluating the articles; efforts were made to contact the authors for clarification.

**Table 2:** Summary of descriptive characteristics of finally selected studies.

*Author Year*	*Article characteristics*	*Participants demographics and characteristics*:	*Method details*			*Main results*
	Study design Aim of the study	No Country Age (mean±SD) Sex	Sense of coherence scale Version of scale *SOC-13 Modified SOC-13* Language Rating scale Cronbach’s α Participants’ SOC (mean±SD)	Oral health behaviors outcome measure	*Statistics analysis method Sampling*	
**Ayo-Yusuf 2008**	Longitudinal Investigated the association between adolescents’ sense of coherence (SOC) and their tooth-brushing behaviour	1025 South Africa 14.4±1.5 Males 47.2% Females 52.8%	SOC-13 English* Seven-point Likert-type 0.63 26.3 (7.2)**	Tooth brushing frequency	Chi-square *t*-tests Step-wise multiple logistic Regression Two-Stage random cluster sampling	Adding baseline intention state to a multivariate model attenuated the influence of baseline SOC to a statistically insignificant level. However, increasing within subject SOC changes (P < 0.01) remained associated with the transition to twice-daily tooth brushing.
**Bernabe et al. 2009**	Cross-sectional Assessed the associations between SOC and childhood socio-economic status with adult oral health-related behaviors	5,399 Finland 49.60±12.78 Males 49.2% Females 50.8%	Modified SOC-13*** Finnish Seven-point Likert-type 0.85 5.48±0.81****	Dental attendance Tooth brushing frequency Dietary habits Smoking habits	Binary logistic regression analysis Two-Stage stratified cluster sampling	SOC was significantly associated with the four oral health-related behaviors. (P < 0.006) Interaction among income, SOC, and gender was statistically significant for dental attendance and tooth brushing frequency (P = 0.042 and 0.001, respectively).
**Da Silva et al. 2011**	Cross-sectional Investigated the relationship of low-socioeconomic status mother’s SOC and their child’s utilization of dental care services	190 Brazil 11.6± 0.95 Girls 56.3%, Boys 43.7%	SOC-13 Portuguese Five-point Likert-type 0.78 47.9± SD = 6.82	Dental attendance	Multivariate logistic regression analysis Convenience sampling	Children whose mothers had higher levels of SOC were more likely to utilize dental care services (P < 0.05) and visit a dentist mainly for check-ups (except for dental treatment) (P < 0.05) than those whose mothers had lower levels of SOC.
**Dorri *et al*. 2010**	Cross-sectional Assessed the association between SOC and tooth brushing behaviors in adolescents	911 Iran 12.42±0.79 Males 59.2% Females 40.8%	SOC-13 Persian Seven-point Likert-type 0.87 48.6 SD±10.7	Tooth brushing frequency:	Binary (multivariate) logistic regression analysis Two-Stage stratified cluster sampling	Higher SOC scores were significantly associated with more frequent tooth brushing behaviors (p<0.01). The association was significant only for girls (p<0.02). However, the interaction between sex and SOC was not significant. (p<0.56) Boys had a significantly stronger SOC than girls. (p<0.04)
**Freire *et al*. 2001**	Cross-sectional Assessed the relationship between adolescents’ sense of coherence (SOC) and oral health	664 Brazil 15 Males 48.9% Females 51.80%	SOC-13 Portuguese Seven-point Likert-type 0.81 57.5	Dental attendance Tooth brushing frequency Dietary habits Fluoride use	Multiple logistic regression analysis Polytomous ordered regression analysis Stratified random sampling	Adolescents with higher SOC were more likely to visit for mainly check-ups compared with those with lower SOC. (p<0.05) Other measures of oral health status and behaviors were not significantly associated with SOC. (p>0.05)
**Freire *et al*. 2002**	Cross-sectional Studied the relationship between mothers’ SOC and their adolescent children’s oral health	664 Brazil 40.1±5.3 Females 100%	SOC-13 Portuguese Seven-point Likert-type—63.9±13.4	Dental attendance Tooth brushing frequency Dietary habits	Multiple logistic regression analysis Polytomous ordered regression analysis Stratified random sampling	Adolescents whose mothers had significantly higher levels of SOC score were less likely to visit the dentist mainly when in trouble than those whose mothers had lower levels of SOC. (p = 0.001) Mothers' SOC was associated with their children's pattern of dental attendance even after adjustment for social class and gender.
**Lindmark *et al*. 2011**	Cross-sectional Investigated the relationship between SOC, oral health–related behavior and knowledge of and attitudes towards oral health	525 Sweden 20–80***** ^1^ Males 49.7% Females 50.3%	SOC-13 Swedish Seven-point Likert-type 0.86 ******	Dental attendance Tooth cleaning habits (Tooth brushing and proximal cleaning frequencies) Dietary habits Smoking habits	Student’s *t*-test One-way ANOVA Tukey test Multivariate logistic regression analysis Stratified random sampling	Individuals with higher total mean SOC scores and subcomponent scores were statistically significantly associated with fewer sweet drinks and a lower frequency of snacks and drinks between meals, compared with individuals with lower total mean SOC scores. (p<0.01) In the bivariate analysis, total SOC was not significantly associated with toothbrushing twice a day or more. Regular dental visiting and smoking habits also did not display any statistically significant relationship with SOC in this study.
**Peker *et al*. 2012**	Cross-sectional Examined the associations between health practices and SOC among dental students at Istanbul University	566 Turkey 21.05±1.62 Males 45.2% Females 54.8%	SOC-13 Turkish Seven-point Likert-type 0.75 56.89±10.68	Dental attendance Tooth brushing frequency Use of dental floss Dietary habits Smoking habits	*t*-test Chi-square test Binary multiple logistic regression Convenience sampling	Students with a strong SOC reported brushing their teeth more frequently (p = 0.008), sugar intake between meals less frequently (p = 0.009), and smoking less frequently (p<0.001) than those with a low SOC. (p<0.05) Participants’ age and sex were not significantly associated with their SOC. (p = 0.24 and p = 0.65 respectively)
**Qiu *et al*. 2013**	Cross-sectional Studied the relationship between caregiver’s SOC and oral health-related behaviors of 5-year-old children	1332 China—Mothers 85.7% Fathers 5.4% Grandparents 8.9%	SOC-13 Chinese Seven-point Likert-type scale 0.86 61.1±10.5	Dental attendance Tooth brushing frequency Dietary habits	*t-* test Chi-square Multiple logistic regression analysis Two-stage stratified cluster sampling	There was no statistically significant difference in the total SOC scores among the different caregivers (p = 0.065). (significant level: p<0.05) No association was found between the children’s sugary snack intake and the mother’s or the father’s SOC. (p<0.05) 8.9% of the children whose grandparents (as caregivers) had significantly higher SOC scores had a lower frequency of sugary snack intake (p = 0.008)

* The original English questionnaires were translated into two local languages, namely Afrikaans and Sepedi for use with a few learners who were not proficient in English; otherwise the surveys were conducted in English.

** Although all the items of the SOC-13 loaded on three factors, the original three-factor structure of the SOC-13 could not be replicated in this adolescent population. only six out of 13 items were replicated for this population; however, the internal consistency coefficient was similar to that of the SOC-13 when comparing them as a unidimensional scale.

***These studies employed an abbreviated form of SOC-13 scale by removing one item to provide equal number of 4 items to measure three constructs of SOC.

**** This study reported SOC score on a 7-point range.

***** Participants have a wide age range which categorized into groups: *Participants were classified into of the following age groups*: *20*, *30*, *40*, *50*, *60*, *70 and 80 years of age*. *No mean for total or each age group has provided*. *The age was classified into four categories*, *30–39 years old*, *40–49 years old*, *50–59 years old and 60–64 years old*. *No mean for total or each age group has provided*. *The age was classified into four categories 40–49*, *50–59*, *60–69*, *and 70–80*. *No mean for total or each age group has provided*.

****** For each behavior SOC was mentioned separately. Refer to table 2 of the article Lindmark et al., 2011 [30].

### Risk of bias in individual studies

The assessment of methodological quality was performed by the same three researchers using the Newcastle-Ottawa Scale (NOS) for cohort studies and modified Newcastle-Ottawa Scale for cross-sectional studies. [25, 26, 27] For the cohort studies, a quality score was calculated based on three categories: group selection (four items), comparability between groups (one item), and outcome and exposure assessment (3 items). A maximum of one point could be awarded for each item in the group selection and outcome and exposure assessment categories. A maximum of two points could be awarded for comparability. Thus, the maximum score was nine points and represented the highest methodological quality. For the cross-sectional studies, the score was calculated based on the same three categories. However, those categories had a different number of items: group selection (four items), comparability (one item), and outcome and exposure assessment (one item). Therefore, the maximum score was seven points and also indicated the highest methodological quality. Studies were considered high quality if they were scored above median: five points for cohort studies and four points for cross-sectional studies [27] Disagreement between the reviewers was discussed until consensus was reached.

### Synthesis of the results

Findings were evaluated in a descriptive manner based on the information provided by each of the included studies. A meta-analysis was not conducted due to the heterogeneity across the studies.

## Results

### Study selection

**Fig 1:**
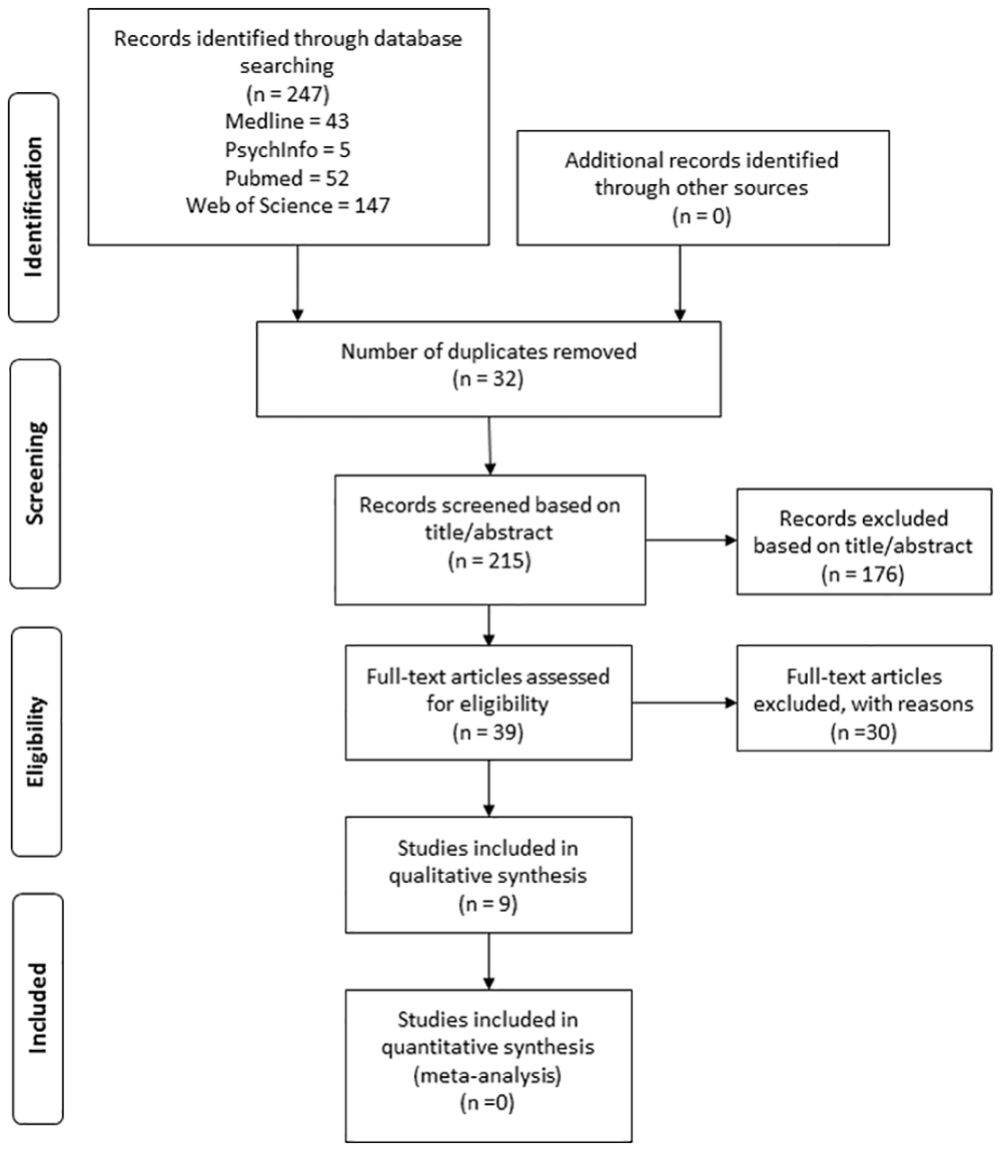
Flow diagram of data search according to PRISMA [24].

The selection process of articles included in this study is presented in the flow chart (Fig 1). A total of 247 records were identified through online searching in the four databases, 46 of which were duplicates. By removing the duplicates, 215 records were screened based on title/abstract. A total of 176 studies were excluded following abstract/title assessment. Therefore, only 39 references were subsequently selected for a full-text analysis.

Of the total 39 full-text articles retrieved and reviewed, 30 studies were later excluded because they were reviews of other studies (6 articles), shared the same population (data source) and outcome variables with another larger study included in this review (7 articles), or their objectives did not meet our inclusion criteria (17 articles); (for instance, they examined the association between SOC and oral health status without evaluating the impact of SOC on oral health behaviors). S2 Appendix displays a summary of the excluded papers and the reasons for their exclusion.

### Study characteristics

The included studies were eight cross-sectional studies and one longitudinal study, published between 2001 and 2013, and conducted in Brazil, [16, 28, 21] China, [18] Iran, [19] Sweden, [22] South Africa, [29] Finland, [30] and Turkey. [31] All selected articles were written in English.

#### Sample characteristics

Sample size ranged from 190 to 5,399 participants. 8 out of 9 reviewed articles involved samples of over 500 participants, and also half of those studied on over 1,000 participants (Table 2). One study employed the available national representative data provided by the National Public Health Institute of Finland. [18] Three studies used two-stage stratified cluster sampling, [18, 28, 28] four studies used stratified random sampling [16, 21, 22, 30] and two used convenience sampling. [28, 31]

Six studies assessed the association between SOC and different oral health behaviors in individuals aged 17 and older. [18, 30, 31] Three studies examined this association in adolescents, between 11 and 16 years. [16, 19, 29] The relationship between mothers’ SOC and their adolescent children’s oral health behaviors was evaluated in two other reports. [21, 28] Finally, one study investigated the correlation between caregivers’ SOC and their preschoolers’ oral health behaviors. [22]

#### Statistical analysis used

For the data analysis, 8 of the 9 studies used bivariate tests as a first step in the analysis strategy in order to test the association between SOC and oral health-related behaviors. The applied methods were t-test, [22, 30, 31] chi-square, [18, 22, 30, 31] simple unadjusted regression analysis, [16, 22], and unadjusted association. [28] One study used descriptive statistics and fit models instead of bivariate test. [19]

In the second step, multivariate analysis was performed in all studies to examine the association of SOC, controlling variables and oral health-related behaviors. Two studies applied binary logistic regression adjusted for gender, age, marital status and urbanization [18]; sex and father education [19]; gender and age [30]. Five studies used multiple logistic regression model adjusted for sex and social class [16, 21]; oral health knowledge and attitude [22]; maternal schooling, marital status, familial income, type of bathroom, and water supply [28]; age [29]. And one study used unadjusted binary logistic regression analysis. [31]

#### Version of SOC questionnaire and reliability

The original questionnaire developed by Antonovsky (1987) contains 29 items named SOC-29 scale (with an average Cronbach’s α of 0.88) [3, 32] that was later shortened into 13 items namely SOC-13 with acceptable reliability (with an average Cronbach’s α of 0.82). [32] All selected studies employed SOC-13 scale. One study [18] used an abbreviated form of the Finnish SOC-13 scale by removing one item to provide equal number of 4 items to measure three constructs of SOC including comprehensibility, manageability, and meaningfulness.

Cronbach’s alpha, used as the measure of internal consistency, ranged from 0.63 to 0.87. One study used the original English version of the questionnaire [29] while other studies used the questionnaire translated and validated into Finnish [18], Turkish [31], Persian [19], Portuguese [16, 21, 28], Chinese [22] and Swedish [30] languages.

For measuring the SOC, seven studies sum up the scores given to each item in the questionnaires in order to calculate SOC mean and standard deviation. [16, 19, 21, 22, 28, 30, 31] One study grouped participants into weak, moderate and strong based on their SOC scores and then in the logistic regression, the model was tested with and without SOC. [18] In the study done in adolescents, [29] only six out of 13 items were replicated for this population; however, the internal consistency coefficient was similar to that of the SOC-13 when comparing them as a unidimensional scale. All studies employed seven-point Likert scale, except for one [28] with a five-point Likert scale.

### Oral health behaviors and SOC

#### Tooth cleaning

Among seven studies [16, 18, 19, 21, 22, 30, 31] examining the association between SOC and tooth brushing behavior, five studies [18, 19, 31] reported a significant association meaning that individuals with a strong SOC were more likely to brush their teeth twice or more per day compared with those who had lower levels of SOC. In one study [30] this association was significant for only one subcomponent of SOC, meaningfulness. The correlation between SOC and tooth flossing behavior was investigated in two studies. [30, 31] No significant association was reported in these studies.

#### Fluoride usage

Three articles assessed the influence of participants’ SOC on their utilization of fluoride products (e.g., mouthwash and gel) and no significant association was reported by any of them. [16, 21, 31]

#### Dietary habits

Six studies aimed to assess the impact of SOC on dietary habits [16, 18, 21, 22, 30, 31], among which, three [18, 30, 31] reported that individuals with a strong SOC were less likely to consume sugar between meals than those with a poor SOC. One of these studies [31] found that among different oral health behaviors, low frequency of between-meal sugar intake was the most important indicator of strong SOC. In another study [22], no correlation was found between children’s sugar snaking and their parents’ SOC; however, among 8.9% of children whose grandparents were their caregivers lower sugar intake observed in those whose grandparents had stronger SOC (p = 0.008).

#### Dental attendance

Among seven papers [16, 18, 21, 22, 28, 30, 31] explored the correlation between SOC score and pattern of dental attendance, four studies [16, 18, 21, 22] reported that individuals with a strong SOC were more likely to visit dentists regularly for check-ups. Two of the studies [21, 28] investigated the association between mothers’ SOC and their children’s regular dental visits reported positive significant association.

#### Smoking habits

The relationship between SOC and smoking was investigated in three papers [18, 30, 31], two of them [18, 31] found a significant correlation indicating that SOC has a positive association with less frequent smoking. One study [30] reported a significant association for only one subcomponent of SOC, meaningfulness.

### Risk of bias within studies

For cross-sectional studies, the methodological quality scores range from three to six points (maximum of seven). [16, 18, 19, 21, 22, 28, 30, 31] The cohort study scored seven points (maximum of nine). [29] The critical appraisal details are presented in Tables 3 and 4. Seven out of the eight included cross-sectional studies scored 4 points and were considered high quality studies. The cohort study was also considered a high quality study.

**Table 3:** New Castle Ottawa (NOS) Quality Assessment [26]*.

	Bernabe et al. 2009	Da Silva et al. 2011	Dorri et al. 2010	Freire et al. 2001	Freire et al. 2002	Lindmark et al. 2011	Peker et al. 2012	Qiu et al. 2013
**Sample selection Criteria (maximum of 4 stars)**								
1) Representativeness of the sample: a) Truly representative of the average in the target population* (all subjects or random sampling); b) Somewhat representative of the average in the target population* (non-random sampling; c) Selected group of patients; d) No description of the sampling strategy	a*	b*	a*	a*	a*	a*	b*	a*
2) Sample size: a) Justified and satisfactory*; b) Not justified.	*	*	*	*	*	b	b	*
3) Non-respondents: a) Comparability between respondents and non-respondents characteristics is established, and the response rate is satisfactory*; b) The response rate is unsatisfactory, or the comparability between respondents and non-respondents is unsatisfactory; c) No description of the response rate or the characteristics of the responders and the non-responders.	c	c	c	c	c	b	c	a
4) Measurement of the sense of coherence: a) Validated measurement tool*; b) Non-validated measurement tool; c) No description of the measurement tool.	a*	a*	a*	b	b	b	a*	a*
**Comparability (Maximum 2 stars)**								
1) Control for confounders a) Participant’s SOC adjusted for one confounder *; b) Participant’s SOC adjusted for two or more confounders **; c) No description related to the adjustment analysis for confounding factors	**	**	**	**	**	**	c	**
**Outcome: (Maximum 1 star)**								
1) Assessment of the outcome from participants: a) Self-report*; b) No description.	a*	a*	a*	a*	a*	a*	a*	a*
**Summary score (maximum of 7 stars)**	6	6	6	5	5	4	3	6

*Note: NOS adapted for cross-sectional studies.

A study can be awarded a maximum of one star (representing “yes”) for each numbered item within the Selection and Outcome categories. A maximum of two stars can be given for Comparability. [27,28]

**Table 4:** Quality assessment of included cohort studies based on the Newcastle-Ottawa scale. [25]

Author	Selection*				Comparability**	Outcome***			Score****
	Representativeness of the exposed cohort^1^	Selection of the non-exposed cohort^2^	Ascertainment of exposure^3^	Demonstration that outcome of interest was not presented at start of study^4^	Comparability of cohorts on the basis of the design or analysis^5^	Assessment of outcome^6^	Was follow-up long enough for outcomes to occur?^7^	Adequacy of follow-up of cohorts^8^	
**Ayo-Yusuf *et al*.**	a*	a*	b*	*	**	c	*	c	7

*a maximum of 1 point for each item

**a maximum of 2 points for each item

***a maximum of 1 point for each item

****a maximum of 9 points

^1^ a) truly representative of the average individuals in the community *, b) somewhat representative of the average individuals in the community *, c) selected group of users, d) no description of the derivation of cohort

^2^ a) drawn from the same community as the exposed group *, b) drawn from a different source, c) no description of the derivation of the non-exposed-group

^3^ a) secure record *, b) structured interview or questionnaire *, c) written self reports, d) no description

^4^ a) yes *, b) no

^5^ a) study control for one confounding variable *, b) study control for 2 or more confounding variables **

^6^ a) independent blind assessment *, b) record linkage *, c) self reports d) no description

^7^ a) yes (select an adequate follow up period for outcome of interest *, b) no

^8^ a) complete follow up—all subjects accounted for *, b) subjects lost to follow up are unlikely to introduce bias—≤20% loss or ≥80% follow up, or description provided of those lost *, c) ≥20% loss or ≤80% follow up, or no description of those lost, d) no statement

## Discussion

There is compelling evidence to support that positive health behaviors could be influenced by psycho-social factors. [3] SOC is a psychosocial determinant of peoples’ health behavior. [6] It appears that individuals with a strong SOC may be more predisposed to a healthy lifestyle and more likely to respond to health-related advice as compared to their counterparts with a weak SOC. [30] In the oral health domain, several studies attempted to investigate the association between individual’s SOC and performance of oral health behaviors such as regular dental check-ups, tooth brushing, and healthy dietary habits. The studies arrived at distinctly different conclusions. Therefore, given the importance of SOC in performing healthy behaviors, this systematic review looked to integrate the research findings with evidence on the impact of SOC on important components of oral health-related behaviors. This topic is particularly important because SOC is considered as a potential theoretical framework to study and better understand oral health behaviors. [28]

In eight of the included studies in this systematic review [16, 19, 21, 22, 28–31], SOC was assessed using a 13-item scale, which was also the most frequently used version of the questionnaire in 54% of the studies included in another systematic review. [32] This version has been validated and translated into different languages. [33] The Cronbach’s alpha of included studies in our review ranged from 0.63 to 0.87, which is in the same range as other systematic reviews of studies using SOC-13 [(0.70 to 0.92) and (0.55 to 0.87)]. [32, 33] For scoring the SOC scale, in some studies instead of summing up the score assigned to each item. [3] SOC was reported on a 7-point range in one study [18] which was different from the scoring method originally proposed by Antonovsky. [3] For this reason, we could not retrieve the mean and standard deviation of SOC and compare them with other studies, which resulted in inconsistency in reporting and comparing the results of this review. In one study, [18] SOC measured using SOC-12, an alternative version of SOC-13, scale. This modified version has been used in several health [8, 33] and oral health studies [34, 35] and showed a reasonable validity and internal consistency. [35]

A positive significant association was found between SOC and healthy diet in adults [18, 31] while in adolescents, this association was not significant. [16] The lack of association may be a result of including a young age group, when oral health behaviors are more likely to be influenced by the parents than arise from the adolescents themselves. However, the association between parental SOC and their adolescents’ healthy dietary habits and practices was found not significant in another study. [21] These results may suggest that neither adolescents’ SOC nor their parents’ SOC influence dietary habits in adolescents. The inconsistency in questions used to measure dietary habits or varying methods/scales and cut-off points by which sugar consumption frequency was measured across the studies could also explain the conflicting results. For instance, daily sugar-intake frequency was measured with answers “Less often than daily” and “On a daily basis”; [18] however, this question was answered through two different items including “None to once” and “Twice or more” in another study. [16].

SOC has received significant empirical research support as a determinant of tooth brushing behavior in adults. [18, 30, 31] In adolescents, there is inconsistency in the results of different studies. While a significant association was reported between stronger SOC and more frequent tooth brushing among Iranian adolescents [19], such an association was not found among 15-year old Brazilian adolescents [16]. One reason for this inconsistency maybe because in adolescents tooth brushing behavior is to some extent influenced by parents and personal sense of coherence is relatively unstable and under development [16, 21]. The second reason may relate to the cut-off point for dichotomizing tooth brushing frequency. In the Brazilian study, [16] this cut off point was three times a day while in the study carried out with Iranian adolescents, [19] the cut off was twice a day. The third possible reason for the inconsistency is that this association may be influenced by cultural differences. [19].

The association between SOC and pattern of dental attendance was investigated on two different levels:

### Adults’ SOC and dental attendance pattern

A positive significant association between SOC and adherence to regular dental visits in adults was observed in two studies. [16, 18] Whereas this association was reported insignificant in another study in which participants’ SOC could not predict their dental attendance pattern. [30] This inconsistency maybe related to the type of questions applied and differences in dental health systems and policies in the various countries, such as the use of a recall system *vs*. dental attendance being based on individual’s initiative. [30] These results support the idea that in adults, those with a high SOC were more likely to visit their dentists regularly for check-ups. [18] According to the SOC concept proposed by Antonovsky, [3] individuals with higher SOC face their daily life challenges in a manner that they are more manageable and predictable. Therefore, attending routine checkups maybe more feasible for these people compared to those with a lower SOC.

### Mothers’ SOC and their children/adolescents’ dental attendance pattern

Increased attention has been given to the impact of psychological aspect of the family environment on children’s oral health. [36] Parental SOC has already been shown to be associated with their children’s oral health status. [9, 36, 37] For instance, mothers have a significant role in the utilization of dental services for children. [38] Parents’ difficulties in dealing with daily problems were also reported to be the main reason for no-shows for their children’s dental check-ups, even when the dental service was available, affordable, or free of charge for them. [39] These findings verify the results from our review that mothers’ SOC was found significantly associated with their children’s dental attendance pattern [21, 28] even after adjusting for socioeconomic [28] and social class [21] variables. In other words, mothers with a strong SOC are more capable of maintaining their children’s oral health including visiting the dentist for check-ups even if they are from a lower socioeconomic class. [28].

SOC was negatively associated with smoking habit in three Brazilian studies [18, 31] and shown to have a protective role against smoking. [31] This result supports the previous reports that people with a poor SOC are more likely to smoke while they are involved in a stressful situation and feel that they are unable to cope with them. [40, 41] However, a study carried out in Switzerland [30] failed to find a similar association between SOC and smoking. One possible reason could be attributed to cultural differences between Brazilian and Switzerland populations.

### Limitations

Some methodological limitations are identified in this systematic review. The first limitation is that almost all selected studies were cross-sectional. The cross-sectional design did not explain causation and changes over time in SOC, specifically regarding the influence of demographic and socio-economic factors on it. [31] The second limitation was the lack of a validated risk of bias assessment tool to measure the quality of the included studies. Although we used the Newcastle-Ottawa Scale methodological assessment tool, to our knowledge, no validated checklist have been developed to assess bias risk within observational studies.

### Recommendations for future research

There is a need for further longitudinal research to shed light on this association through a life course approach. In addition, improving SOC in the early stages of life while it is under development may have significant impact on an individual’s life course and well-being. Consequently, it could be considered as a proactive way to improve the effectiveness of oral health promotion and intervention programs. [42] For instance, an intervention has been developed to enhance individual's SOC by improving its components. The intervention was based on literature searches, guidelines from educators and previous work about SOC. It involved the approach of children and their mothers through classroom activities and healthy school programs with brainstorming, planning, implementation and evaluation. [42] The improved SOC following such an intervention may promote oral health since individuals with a strengthened SOC select more favorable oral behaviors [18] and cope better with stress. This can also lead to lower biological effects, such as oral diseases [43] and a better oral health-related quality of life. [44]

Further research is also required to see if it is possible to introduce SOC as a psychological construct that could be considered in oral health models. Regarding the impact of mothers’ SOC on their children’s oral health practices, it would be worthy to find strategies to structure and develop mothers’ SOC with the aim to improve their children’s oral health behaviors in particular taking their children for preventive dental checkups.

## Conclusions

- A cross-sectional association was found between SOC and oral health-related behaviors. A stronger SOC was associated with more favorable behaviors of tooth brushing frequency, daily smoking, and dental attendance.
- Mothers’ SOC can influence children’s oral health practices in particular their pattern of preventive dental attendance.

## Supporting Information

S1 Appendix Search strategies and results from different electronic databases. (DOCX)

S2 Appendix Excluded articles and the reasons for their exclusion. (DOCX)

S3 Appendix PRISMA 2009 Checklist (Liberati et al., 2009). (DOCX)
