# Supplementary material for: Impact of Sense of Coherence on Oral Health Behaviors: A Systematic Review
Source: PLoS One. 2015 Aug 14;10(8):e0133918. doi: 10.1371/journal.pone.0133918 (PMC4537196; doi:10.1371/journal.pone.0133918)
Supplement: S1 Appendix — (DOCX) [file pone.0133918.s001.docx]

**S1 Appendix. Search strategies and results from different electronic databases**

| **Database** | **Keywords** | **Results** |
| --- | --- | --- |
| PubMed (1966 to April  2015, week 1) | ((((("sense of coherence"[All Fields] OR "sense of coherence scale"[All Fields]) OR "salutogenic model"[All Fields]) OR "salutogenic approach"[All Fields]) OR "salutogenic theory"[All Fields]) OR "salutogenic concept"[All Fields]) AND (((((((("Oral Health"[All Fields] OR "oral hygiene"[All Fields]) OR "tooth brushing"[All Fields]) OR "dental attendance"[All Fields]) OR "dental education"[All Fields]) OR "dental"[All Fields]) OR "dentistry"[All Fields]) OR ("dental caries"[MeSH Terms] OR ("dental"[All Fields] AND "caries"[All Fields]) OR "dental caries"[All Fields] OR "caries"[All Fields])) OR "oral habit"[All Fields]) | 52 |
| Ovid MEDLINE(R)  (1946 to April 2015 Week 1) | exp "Sense of Coherence" OR  "sense of coherence".ti,ab. OR  “(salutogen* adj2 (model* or concept*)).mp.” AND “exp Oral Health/ or exp Oral Hygiene/ or exp Periodontal Diseases/ or exp Dental Caries/ or exp Dental Health Surveys/ or exp Tooth Diseases/ or exp Health Education, Dental” OR ((oral or dental) adj2 (health or hygiene or medicine or care)).mp. OR “(dental adj2 caries).ti,ab. OR “((tooth or periodontal) adj2 disease*).ti,ab.” | 43 |
| ISI Web of Science  (1965 to April 1, 2015) | Topic=(sense of coherence OR salutogenic OR salutogenesis ) AND (TS=oral AND (health OR hygiene) OR TS=(dental OR dentist* OR caries OR cavities OR gingivitis))  Timespan=1985-2014. Databases=SCI-EXPANDED. Lemmatization=On | 147 |
| PsychInfo (1980 to April 2015) | ((((("sense of coherence"[All Fields] OR "sense of coherence scale"[All Fields]) OR "salutogenic model"[All Fields]) OR "salutogenic approach"[All Fields]) OR "salutogenic theory"[All Fields]) OR "salutogenic concept"[All Fields]) AND (((((((("Oral Health"[All Fields] OR "oral hygiene"[All Fields]) OR "tooth brushing"[All Fields]) OR "dental attendance"[All Fields]) OR "dental education"[All Fields]) OR "dental"[All Fields]) OR "dentistry"[All Fields]) OR ("dental caries"[MeSH Terms] OR ("dental"[All Fields] AND "caries"[All Fields]) OR "dental caries"[All Fields] OR "caries"[All Fields])) OR "oral habit"[All Fields]) | 5 |
| Total databases searches |  | 247 |
| Duplicates |  | 32 |
| Final |  | 215 |
