# Supplementary material for: Impact of Sense of Coherence on Oral Health Behaviors: A Systematic Review
Source: PLoS One. 2015 Aug 14;10(8):e0133918. doi: 10.1371/journal.pone.0133918 (PMC4537196; doi:10.1371/journal.pone.0133918)
Supplement: S2 Appendix — (DOCX) [file pone.0133918.s002.docx]

| ***Authors/ Year*** | ***Resason for exclusion*** |
| --- | --- |
| Ayo-Yusuf , et al., 2008 | 3 |
| Baker, et al., 2010 | 1 |
| Bernabe, et al., 2012 | 3 |
| Bernabe, et al., 2009 | 2 |
| Bernabe, et al., 2012 | 2 |
| Bernabe, et al., 2010 | 2 |
| Boman, et al., 2012 | 3 |
| Bonanato, 2009 | 3 |
| Chang, et al., 2010 | 3 |
| Dorrir, et al., 2010 | 2 |
| Domitrescu, et al., 2010 | 3 |
| Emami, et al., 2010 | 3 |
| Holister, et al., 2004 | 1 |
| Johahanson, et al., 2012 | 3 |
| Karla, et al., 2013 | 3 |
| Lindmark, et al., 2011 | 3 |
| Lindmark, et al., 2011 | 3 |
| Mattila, et al., 2011 | 3 |
| Morita, et al., 2007 | 3 |
| Morita, et al., 2008 | 3 |
| Nammontri, et al., 2013 | 3 |
| Rivera, et al., 2013 | 1 |
| Savolainen, et al., 2004 | 2 |
| Savolainen, et al., 2005 | 3 |
| Savolainen, et al., 2005 | 2 |
| Savolainen, et al., 2009 | 2 |
| Silva, et al., 2008 | 1 |
| Sirkka, et al., 2013 | 3 |
| Slade, et al., 2013 | 1 |
| Watt, 2002 | 1 |
| 1. Not original study (review stidies); 2. Shared exactly the same population and outcome variables with another article reported in this review; 3. The objective of study did not meet the inclusion criteria. (For instance, it examined the associatuion between SOC and oral health status without evaluating the impact of SOC on oral health behaviors.) | |

**S2 Appendix. Excluded articles and the reasons for their exclusion**
